# Toxicity and cosmesis outcomes after single fraction partial breast irradiation in early stage breast cancer

**DOI:** 10.1186/1748-717X-6-155

**Published:** 2011-11-11

**Authors:** Paola Pinnarò, Stefano Arcangeli, Carolina Giordano, Giorgio Arcangeli, Fabrizio Ambesi Impiombato, Valentina Pinzi, Giuseppe Iaccarino, Antonella Soriani, Valeria Landoni, Lidia Strigari

**Affiliations:** 1 Department of Radiation Oncology, Regina Elena National Cancer Institute, Rome, Italy; 2 Laboratory of Medical Physics and Expert Systems, Regina Elena National Cancer Institute, Rome, Italy

## Abstract

**Background:**

To report the clinical outcome after a Single Shot 3D-CRT PBI (SSPBI) in breast cancer patients after conservative surgery (ClinicalTrials.gov Identifier: NCT01316328).

**Methods:**

A dose of 18Gy (in the first 4 patients) and 21Gy (in the remaining 60 patients) was prescribed in a single session and delivered to the index area (i.e. the area of breast including the primary tumor bed and the surrounding tissue) using 3D-CRT with patients in prone position. Acute and late toxicity was assessed using the National Cancer Institute's CTC for Adverse Events. Cosmesis was defined based on modified Harvard criteria. Differences between dosimetric or clinical parameters of patients with/without G2 or more late toxicity or unsatisfactory (poor or fair) cosmetic outcome were evaluated with the Mann-Whitney test. Odds ratios and 95% confidence interval were calculated for cosmesis and fibrosis. Univariate and multivariate analyses(UVA/MVA) were used to determine covariates associated with an increase in fibrosis or fat necrosis rate.

**Results:**

Sixty four patients were enrolled. With a median follow-up of 3 years, G2 and G3 subcutaneous fibrosis was detected in 20(31%) and in 8(13%) patients, and ≥G2 fat necrosis was observed in 2(3%) patients. Good to excellent, fair and poor cosmesis was observed in 38(59%), 23(36%) and 3(5%) patients, respectively. Based on UVA, the breast volume receiving more than 21Gy (V_21_Gy) was found to be a predictor of the ≥G1 or ≥G2 fibrosis/fat necrosis. Based on MVA, V_21_Gy was confirmed as a predictor for ≥G1 fibrosis/fat necrosis, the results correlated as a trend for ≥G2. Cosmesis was correlated with whole breast (WB) mean dose (p = 0.030).

**Conclusion:**

Our choice of a single dose of 21Gy significantly increased the treatment related toxicity. However, this should not discourage novel SSPBI approaches with lower equivalent doses.

## Background

A number of studies [1-3] have reported that most (81%-100%) intra breast tumor recurrences after breast conserving therapy occur in close proximity to the tumor bed, thus providing the rationale for an adjuvant radiotherapy limited to the Index Area, i.e. the area of breast including the primary tumor bed and the surrounding tissue.

In fact, the risk of recurrence outside the operative area has been reported to be similar to the risk of new primary cancer in the contra-lateral breast, usually not irradiated [4]. This new philosophy of Partial Breast Irradiation(PBI) has suggested new clinical and technical radio-therapeutic approaches. It has been theorized that, limiting the dose to smaller volumes, larger radiation fractions can safely be used reducing the overall treatment time.

Thus, Accelerated PBI (APBI) has been promoted in phase I-III trials [5-9] designed to test the feasibility and equivalence compared with standard Whole Breast Irradiation(WBI) in properly selected low-risk early breast cancer patients after breast conserving surgery(BCS).

A number of approaches are now available for the implementation of APBI including: intra-operative radiotherapy (IORT) using electron [4],and 50 kVp X-rays[10], multi-catheter interstitial brachytherapy [11], balloon-based high dose rate (HDR) brachytherapy [12], Pd-103 permanent seed implant [13] and three-dimensional conformal external beam radiotherapy(3D-CRT) [14].

All these techniques, allowing the RT treatment to be completed within 5 days, as opposed to a 5-7 weeks of WBI, might increase the use of BCS, by helping to overcome the "logistical barriers" (age/age morbidity, time, travel difficulties, absence from family/job, costs, waiting list delays for RT or chemotherapy, etc.) and providing more women with this option.

Another advantage of APBI could be a decreased dose to lungs and heart. Several data have emerged about a potential correlation between WBI and the risk of cardiovascular and pulmonary toxicities [15,16], especially in patients receiving cardio-toxic agents [17,18]. A further concern is the increased risk of lung cancer in patients irradiated for breast cancer [16]. Therefore, with a reduction in irradiated breast volume the heart and lungs should receive lower doses, reducing the risk of late toxicity and/or new primary lung cancer.

3D-CRT has several potential advantages, such as the ability to offer the treatment after full pathologic information, decreased operator-dependence, and less financial expenses. In addition, it is not invasive and is widely available in all radiotherapy departments equipped with standard modern facilities (multileaf collimators, CT simulation, dedicated couch, treatment planning system).

Although some studies are currently ongoing, no data on the superiority of a selected technique and/or fractionation schedule has yet been reported. However, caution should be taken on late toxicity of PBI using larger dose/fraction. Therefore, cosmetic outcome and toxicity need to be more fully investigated.

A PBI approach based on Single Shot 3D-CRT PBI (SSPBI) with patients in prone position has been reported by our Institute to be feasible and able to spare heart and lungs [19]. However, the reported follow-up was too short to draw any conclusion regarding radiation induced late toxicity. In this paper we report the acute and late toxicity, including fibrosis and cosmetic outcome, on a larger cohort of patients who underwent SSPBI in prone position, with the aim to describe the dose effect relationship based on the updated radiobiological parameters.

## Methods

### Patients

Eligibility criteria were: Age ≥48 years with a life expectancy of at least 5 years, post-menopausal status, histologically proven, non lobular, adenocarcinoma of the breast, primary tumor ≤ 3 cm, negative surgical margins (≥ 2 mm), negative sentinel nodes or <4 positive axillary nodes, no extra-capsular extension, no previous radiotherapy. Patients with multicentric disease, extended intraductal component (EIC>25%), Paget's disease of the nipple, lobular adenocarcinoma, distant metastases, were not included in this study. The protocol has been approved by the local Ethics and Scientific Committee. All patients were informed and provided a written informed consent.

Patients underwent physical examination and photographs at baseline and once a week for one month after SSPBI, every month for the first 3 months and every six months afterwards. Systematic photographs (one frontal and two profile) were taken under the same patient position.

Acute/late toxicity was assessed using the National Cancer Institute's Common Terminology Criteria for Adverse Events (CTCAE),vers.3.0[20]. Fat necrosis was scored according to the system proposed by Lövely et al.[21].Toxicity was defined as acute/late if it occurred before/after the 6-month post-RT.

Based on modified Harvard criteria[22], cosmesis was defined on a four-point scale (excellent/good/fair/poor), by a physician not involved in the study, comparing pre-RT baseline breast photographs with those taken at the last follow-up. An "Excellent" score indicated that the treated breast was essentially the same (minimal or no difference in size/shape). A "Good" score indicated minimal but identifiable RT-related effects (slight difference in size/shape). A "Fair" score indicated significant readily observable effects (moderate deformity with notable differences in the size/shape). A "Poor" score indicated severe sequelae (marked changes in the appearance involving more than one quarter of the breast).

The local, regional and distant relapse was assessed during follow-up based on clinical and radiological examination. In particular mammography and breast US examinations were done at 6 and 12 months after SSPBI and then once a year.

### Radiotherapy treatment

**Figure 1:**
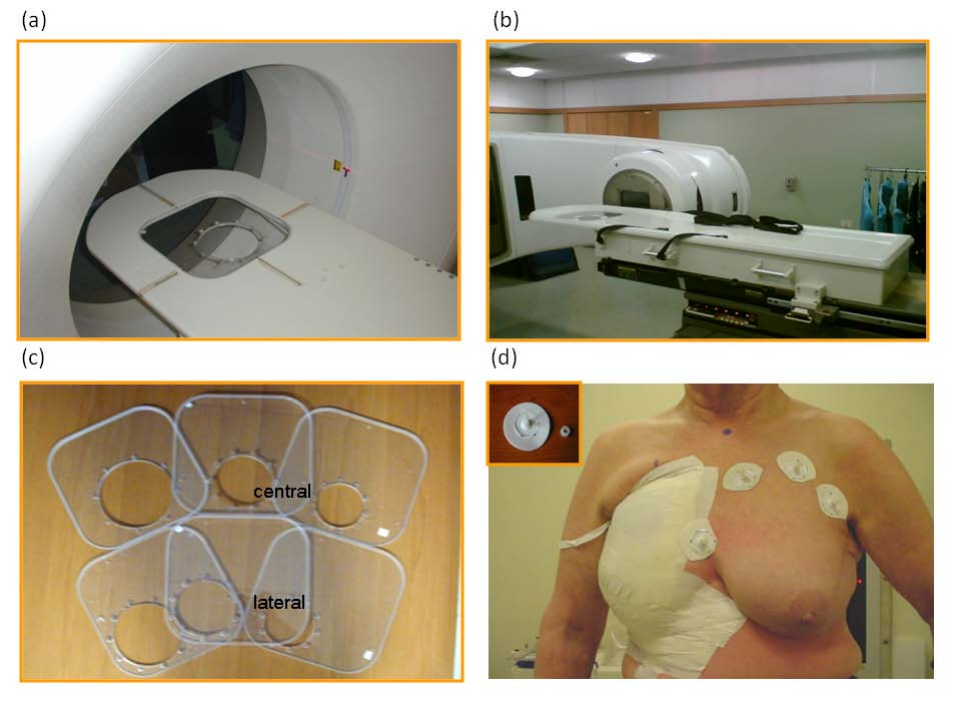
**Positioning device and patient preparation**. Positioning device hooked to the a) CT simulator and b) Varian treatment couch with PMMA frames with a 15 cm diameter circular opening placed inside the table top, c) PMMA frames with different diameters and positions, d)Patient preparation: the contra-lateral breast was bandaged, the pads are glued to the skin by their adhesive area, when the patient lies on the table the plastic bolts are inserted inside the corresponding holes and the breast is fixed to the table by nuts.

Patients underwent the CT simulation and treatment in prone position on a specifically designed home-made couch with a circular aperture, allowing the involved breast to hang from the chest wall and to receive radiation from multiple fields with the gantry in the horizontal position. Adequate immobilization prevents any breast movement related to breathing, enabling precise target volume (PTV) definition and treatment. Technical and physical details of the procedure have been fully described in a previously published paper [19]. Briefly, the patient is in prone position on a particular home-made positioning device that can be coupled both to the CT (Figure 1.a) and treatment couch (Figure 1.b). This device basically consists of a table top with a square opening, with a side of 30 cm, at the center of the proximal side where different square frames can be placed. The frames (Figure 1.c), through which the breast hangs down, have a circular opening of different diameters (11 to 19 cm). The thoracic region around the patient's breast can be fastened to the frame by means of thin circular plastic pads glued to the skin by their adhesive area (Figure 1.d). Polymethyl methacrilate (PMMA) bolts at the center of the other side, can be screwed to the numbered holes drilled along the opening rim, thus ensuring the breast repositioning and steadiness. A similar approach was already tested by Formenti and co-workers [14].

The tumor bed was identified by the metallic clips positioned at the time of surgery. In all other patients the tumor bed was defined according to tumor location in preoperative mammographic images and by the difference in density on CT slices, between the mammary and scar tissue. The tumor bed consisted of the scar tissue formed after the mammary gland flaps were sutured leaving virtually no gap between them. The CTV was finally contoured on every 3 mm CT slices by adding 1.5-2 cm of breast tissue to the tumor bed.

**Figure 2:**
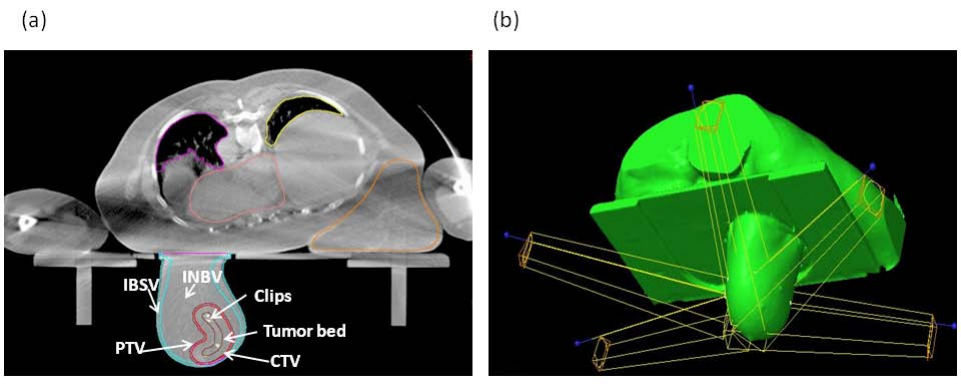
**Contours and clips on CT slices**. a) CT slice with clips, CTV, PTV and OARs, including INBV = ipsilateral normal breast volume, IBSV = ipsilateral breast skin volume. b) fields arrangement.

The PTV was obtained by 3 mm uniform expansion around the CTV and was limited by the skin and the chest wall. The organs at risk (OARs) included the skin, the remaining ipsilateral/contra-lateral breast, ipsilateral lung and heart. The contours of a representative patient are reported in Figure 2.a.

**Figure 3:**
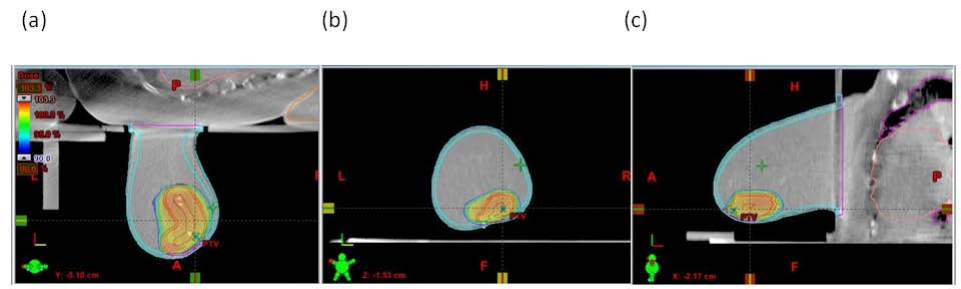
**Dose distribution**. Dose distribution in a representative patient in a) trasversal, b) coronal, c) sagittal plane. PTV outlined in black. Some surgical clips are visible.

**Figure 4:**
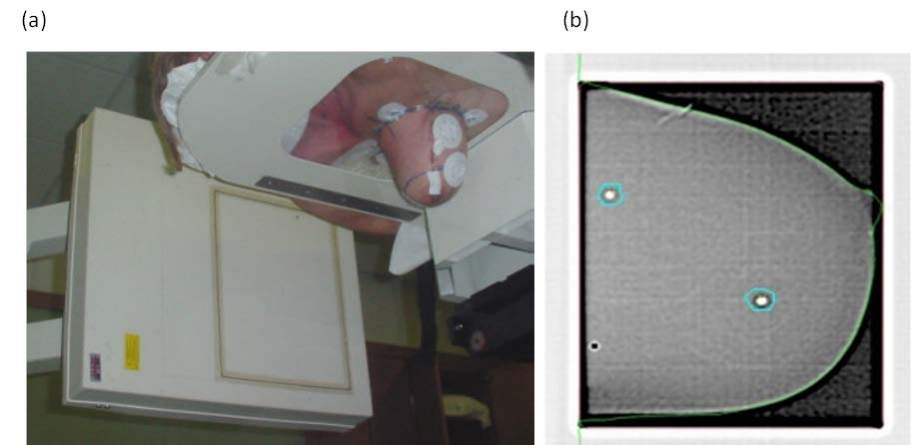
**Treatment setup verification by portal imaging**. Treatment setup verification by portal imaging. a) Image acquired with gantry at 90 degrees. b) portal image matched with the DRR image. The breast correct position is verified by lead marker and breast profile alignment.

3D-CRT treatment plans were developed with the Eclipse v.6.5 (Varian Medical System, Palo Alto, CA) by using five fixed non coplanar beams of 6 MV photons covering 220 degrees (Figure 2.b). Dynamic wedges were used to compensate for tissue thickness variations along the thoracic wall to nipple direction and for the non symmetrical fields arrangement. Dose distribution in a representative patient in a) trasversal, b) coronal, c) sagittal plane is reported in Figure 3. The occurrence of breast motion due to respiration during irradiation was monitored by using electronic portal images by means of Portal Vision aS500 image detector (Varian Medical System) set in cine modality, verifying the position of a lead marker aligned to the exit site of the beam isocenter, before and immediately after the treatment delivery on the image sequences (Figure 4). A dose of 18Gy (in the first 4 patients) or 21Gy (in the remaining 60 patients), normalized to the PTV mean dose, was prescribed in a single session. The treatment time was 5-7 minutes, by using a dose-rate of 600 MU/min.

Additional recommendations included: dose ≥ 50% of the prescribed dose (PD) to ≤50% of the ipsilateral, i.e. normal breast tissue (INBV = WBV-PTV) (V_50%PD_≤50%), and doses as low as possible to the ipsilateral breast skin. The maximum and mean dose to WB, INBV and skin were calculated, as well as, the PTV/WB i.e. the ratio between the PTV and the WB volume. The recorded dosimetric characteristics for WB also include V_XGy_(%/cc), that represents the percentage/absolute volumes receiving more than XGy.

### Radiobiological consideration

The dose of 21Gy in a single fraction was established on the basis of the biologically effective dose (BED) given by: BED=(nd)x(1+d/α/β) [23] where n is the number of fractions, d is the dose/fraction and α/β indicates the response to the dose fractionation. In fact, the standard treatment schedule of 30 fractions of 2Gy within 6 weeks - 50 Gy to the entire breast with photon beams plus a boost of 10 Gy to the tumor bed with electron beams has a corresponding BED of 72 Gy (using α/β = 10 Gy), corresponding to a biologically equivalent single dose of 22.3 Gy. Therefore, a single dose of 21 Gy was considered reasonable to treat the tumor, in agreement with Veronesi et al. [24], who reported different dose levels tested from 10 to 21 Gy without significant side-effects after a median follow-up of 36 months.

### Study design and Statistical analysis

This study was designed as a prospective "single-arm study" having an unsatisfactory (poor or fair) cosmetic outcome not superior to the conventional WBI (50 Gy+boost), maintaining an equivalent local control. The sample size (100 patients) was calculated to estimate the unsatisfactory cosmesis rate or ≥Grade 2 (G2) toxicity (expected to be about 10%) with a standard error of 3%.

Decision rules were drawn up to stop the trial early if ≥15% of patients showed an unsatisfactory cosmesis or ≥G2 toxicity at any point.

For all endpoints, patients were censored at the time of the specific event. Actuarial curves for late toxicity were calculated by the Kaplan-Meier product-limit method and compared by the log-rank test.

Univariate and multivariate analyses (UVA/MVA) were used to determine covariates associated with an increase in ≥G2 fibrosis or fat necrosis rate. Clinical and dosimetric factors tested by univariate analysis included chemotherapy (yes/no), hormone-therapy (yes/no), upper/lower/supero-external quadrant (yes/no), post-operative complications (yes/no), PTV/WB>10% (yes/no), V_18 Gy_>20% (yes/no), V_21 Gy_>66 cc (yes/no), PTV>97 cc (yes/no), WB mean dose>9 Gy (yes/no), skin mean dose>5 Gy (yes/no). To compare groups the median values of dosimetric/volumetric data was taken as cut-off. While in the MVA, dosimetric and volumetric factors were considered as continuous variables.

Odds ratios and 95% confidence interval (CI) were calculated for cosmesis/fibrosis. T-tests for continuous variables were performed. Differences between groups were also evaluated by the Mann-Whitney test. All times were calculated from the day of radiotherapy.

## Results

The study was stopped prematurely before reaching the planned sample size of 100 patients, due to the unexpected unsatisfactory cosmesis rate or ≥G2 toxicity. From March 2006 to January 2008, 64 patients, median age 66 years who underwent BCS and sentinel node biopsy and/or axillary dissection for early breast adenocarcinoma were treated in prone position with an adjuvant SSPBI schedule to the index area. Sixteen patients (25%) received adjuvant, non-concomitant, chemotherapy, consisting of CMF (Cyclophosphamide 600 mg/m2), Methotrexate (40 mg/m2,5-FU 600 mg/m2;d1 and d8;q4 weeks × 6) in 5 patients, or FEC (5-FU 600 mg/m2,Epirubicin 60 mg/m2), Cyclophosphamide (600 mg/m2;d1;q3 weeks × 6) in 5 patients, or EC (Epirubicin 60 mg/m2,Cyclophosphamide 600 mg/m2;d1;q3 weeks × 4) in 1 patient, or EC followed by Docetaxel (100 mg/m2;d1;q3;weeks × 4) in 5 patients. Adjuvant chemotherapy was completed 3/4 weeks before SSPBI except in one patient (who received chemotherapy one-week after). Adjuvant hormone-therapy, with Tamoxifen (6 pts.) or Anastrozole (44 pts.) or Letrozole (8 pts.), if indicated, was given simultaneously with SSPBI. Patient, tumor and treatment related characteristics are listed in Table 1. The median follow-up was 37 months. The main dosimetric and volumetric characteristics are reported in Table 2.

**Table 1:** Main patient characteristics

Age (years)	*median (range)*	66.5 (51-87)
		
**Histology**	*Ductal/others *	55/11
		
**Grading (G)**		
	*1*	13
	*2*	43
	*3*	8
		
**Chemotherapy**		
	*yes/no*	16/48
		
		
**Hormonotherapy**	*yes/no *	58/6
	TAM	6
	A	44
	L	8
		
**Estrogen receptor**		
	*RE+*	58
	*RE-*	6
		
**Progesteron receptor**		
	*RP+*	51
	*RP-*	13
		
**tumor side**	*right/left *	26/38
		
**tumor diameter (mm)**	*median (range)*	12(2-30)
		
**Quadrant**		
	*Upper*	58
	*Lower *	6
		
**Tumor stage**		
	*Tis*	1
	*T1*	53
	*T2*	10
		
**Nodal stage**	*N0/N1*	60/4
		
**Follow-up (months)**	*median (25^th ^- 75^th^)*	37(31 -39)

**Abbreviations:**TAM = tamoxifen; A = anastrozole; L = letrozole

**Table 2:** The main dosimetric and volumetric data observed in our patients' group.

Parameter	Median	Min	Max
CTV volume (cc)	79	15	240
PTV volume (cc)	96	17	290
PTV minimum dose (cGy)	1804	829	1988
PTV mean dose (cGy)	2112	1797	2181
PTV maximum dose (cGy)	2185	1870	2247
Whole breast volume (cc)	892	230	2608
Whole breast mean dose (cGy)	971	436	1411
INBV (cc)	743	205	2441
INBV mean dose (cGy)	740	100	1153
Skin volume (cc)	165	64	385
Skin mean dose (cGy)	544	246	845
V_2.1 Gy _(%)	84	41	99
V_18.0 Gy _(%)	20	5	41
V_21.0 Gy _(%)	9	0	28
V_21.0 Gy _(cc)	67	0	263
PTV/WB	12%	3%	26%

**Abbreviations: **WB = Whole breast; INBV = WB volume -PTV; PTV/WB = ratio between PTV and WB volume; V_XGy_(%) or V_XGy_(cc), that represent the percent of the breast volumes or the volume (cc) receiving more than × Gy, respectively.

### Acute toxicity

Table 3 shows the acute toxicity observed in our cohort. Nineteen (30%) patients complained of a G1 acute pain, which developed only at the end of treatment, likely due to the immobilization position discomfort, and disappeared within 1-2 days. No G2 or more acute pain toxicity was detected.

**Table 3:** Acute and late toxicity observed in our cohort.

Toxicity	G0	G1	G2	G3
Acute pain	*70%*	30%	0%	0%
				
Acute erythema	*67%*	17%	14%	2%

Hyper-pigmentation	*84% *	16%	0%	0%
				
Teleangectasia	*73%*	25%	2%	0%
				
Late pain	*53%*	45%	2%	0%
				
Subcutaneous fibrosis or fat necrosis	*19%*	36%	31%	14%

**(cosmetic outcome)**	***(excellent)***	**(good)**	**(fair)**	**(poor)**
breast appearance	*20%*	39%	36%	5%

G1, G2 and G3 acute erythema was observed in 11 (17%), 9 (14%) and 1 (2%) patients, respectively. A statistically significant correlation was found between the ≥G1 erythema and the skin mean dose (p = 0.008), WB mean dose (p = 0.008), V_18 Gy _(p = 0.009), PTV (p = 0.010) and PTV/WB ratio (p = 0.041). No correlation was found between acute toxicity and chemotherapy.

### Late toxicity

Table 3 shows the late toxicity observed and the cosmetic outcome. The main results of UVA are reported in Table 4. G1 hyperpigmentation was observed in 10 patients (16%) with no correlation with dosimetric/volumetric data. No patient developed G2 or more hyperpigmentation.

**Table 4:** Results based on univariate analysis

Parameters	p-value
Erythema (≥G1vs. G0) against skin mean dose	0.008
Erythema (≥G1vs. G0) against WB mean dose	0.008
Erythema (≥G1vs. G0) against V_18 Gy_	0.009
Erythema (≥G1vs. G0) against PTV	0.010
Erythema (≥G1vs. G0) against PTV/WB	0.041

Telangectasia (≥G1vs. G0) against hormonal therapy	0.0178
Telangectasia (≥G1vs. G0) against V_18Gy _>20%	0.032
Telangectasia (≥G1vs. G0) against V_21Gy_>8%	0.0352
Telangectasia (≥G1vs. G0) against PTV > 97 cc	0.0406
Telangectasia (≥G1vs. G0) against previous acute erythema	0.0132

Late pain (≥G1vs. G0) against the upper quadrant	0.0446

Fibrosis/fat necrosis (≥G1vs. G0) against V_18Gy_>20%	0.0258
Fibrosis/fat necrosis (≥G1vs. G0) against V_21Gy_>66cc	0.00043
Fibrosis/fat necrosis (≥G1vs. G0) against WB mean dose>9Gy	0.0284
Fibrosis/fat necrosis (≥G1vs. G0) against PTV>97cc	0.0133
Fibrosis/fat necrosis (≥G2vs. G0-G1) against V_21Gy_>66cc	0.0033

Cosmesis (good/excellent vs.poor/fair) and WB mean dose >9Gy	0.030
Cosmesis (good/excellent vs.poor/fair) and ≥G2 fibrosis	<0.0012
Cosmesis (good/excellent vs.poor/fair) and G3 fibrosis	0.0003

G1 and G2 telangectasia (Table 3) was observed in 16 (24.5%) and 1 (2%) patients, respectively. Telangectasia was found to be correlated with hormonal therapy (p = 0.0178), V_18Gy _>20% (p = 0.032), V_21Gy_>8% (p = 0.0352), the PTV volume larger than 97 cc (p = 0.0406) and previous acute erythema (p = 0.0132).

From the 64 patients, 29 and 1 patients complained of a G1 and G2 late pain, respectively, the latter likely related to G3 fat necrosis (see later). Late pain (Table 3) was more frequently reported after the irradiation of the lower than the upper quadrant (83% vs.43%, respectively, p = 0.0446).

**Figure 5:**
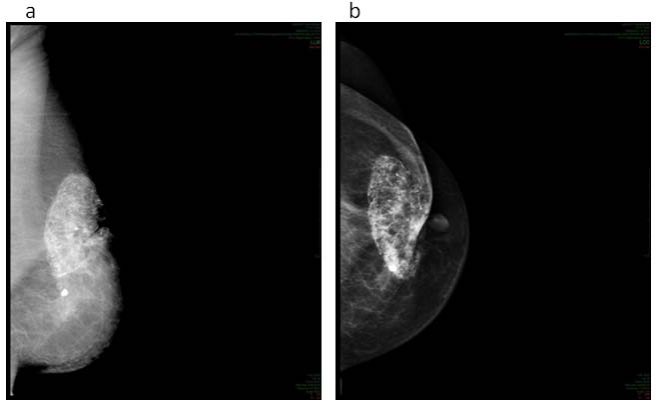
**G3 fat necrosis**. Mammographic image of G3 fat necrosis observed in 1 patient.

Subcutaneous fibrosis was detected in 51(80%) patients. Fibrosis was mild (G1) in 23 (36%), moderate (G2) in 20 (31%) and severe (G3) in 8 (13%) patients. G1,G2 and G3 fat necrosis were observed in 1 (1.5%), 1 (1.5%) and 1 (1.5%) patient, respectively. Figure 5 shows the radiological imaging of G3 fat necrosis.

UVA (Table 4) showed that the ≥G1 fibrosis/fat necrosis correlated with the V_18Gy_>20% (p = 0.0258) or V_21Gy_>66cc (p = 0.00043), the WB mean dose>9Gy (p = 0.0284) and the PTV>97cc (p = 0.0133), while ≥G2 fibrosis/fat necrosis(corresponding to a marked increase of density and firmness on palpation with/without retraction/fixation) correlated to the V_21Gy_>66cc (p = 0.0033).

The ≥G1 fibrosis/fat necrosis odds risk was 4.2 (95%CI:1.1-15.5) when PTV/WB>10%; 15 (95%CI:1.8-125) when V_18Gy_>20% and 5.7 (95%CI:1.4-24) when the WB mean dose was >9Gy. The odds risk of ≥G2 fibrosis/fat necrosis was 3.2 (95%CI:1.1-8.8) when V_21Gy _was over 66cc.

Based on MVA, the ≥G1 or ≥G2 fibrosis/fat necrosis toxicity was statistically (p = 0.02) or as trend (p = 0.09) correlated to the breast volume receiving more than 21Gy. In particular the higher the volume of breast exposed to this threshold dose, the greater the risk of developing a ≥G1 or ≥G2 fibrosis/fat necrosis reaction, with an increment of risk of 0.006 per each cc of breast.

**Figure 6:**
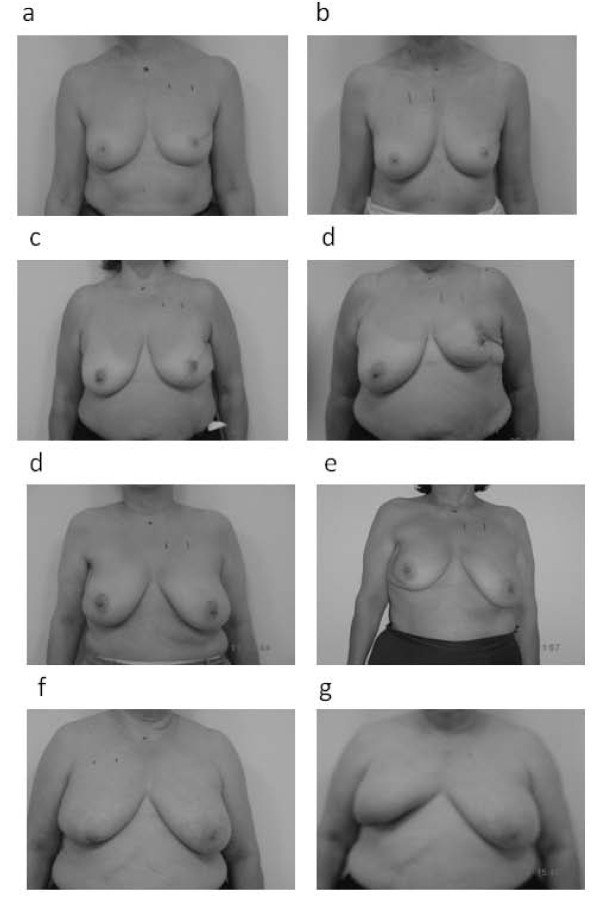
**Photographs of cosmetic outcome**. Photographs a)before and b) after RT of a patient with excellent cosmetic outcome; photographs c),e),g) before and d),f),h) after RT of three patients with poor cosmetic outcome. The cosmetic outcome was defined according to modified Harvard criteria (see text).

The cosmetic outcome was scored in all patients. Good/excellent cosmesis was observed in 38 patients (59%), fair and poor cosmesis in 23 (36%) and 3 (5%) patients, respectively (Table 3). Figure 6 shows a patient with excellent cosmesis along with the only 3 patients with poor cosmesis.

No correlation was found between cosmesis (good/excellent vs.poor/fair) and dosimetric/volumetric data with the exception of WB mean dose >9Gy (p = 0.030). Moreover, a correlation between cosmesis and ≥G2 or G3 fibrosis (P < 0.0012 and 0.0003, respectively) was found. The odds risk analysis confirmed no correlation between cosmesis and clinical/dosimetric or geometric parameters. No correlation was found between late toxicity and chemotherapy data.

### Recurrence

One patient, who met the ASTRO's "cautionary" criteria for APBI, underwent mastectomy for suspected recurrence, histologically confirmed. Of relevance, this patient was free from any grade of subcutaneous fibrosis.

## Discussion

In recent years a number of phase I-II trials have been published with the aim of exploring the feasibility of hypo-fractionation regimens, delivering total doses between 10 to 38.5Gy, assumed to be equivalent to 45-50Gy at 1.8-/2Gy/fraction using an α/β of 10 Gy [24], as a possible alternative to conventional WBI schedules.

Whereas, most initial experiences with APBI using brachytherapy[9,11-13,25-29] have led to optimal results in terms of local control and toxicity, data regarding the acute and late toxicity with external beam PBI are sparse and rather conflicting. By delivering a dose of 38.5Gy in 10 fractions twice-daily to the lumpectomy cavity, some papers showed no or very few local recurrence with excellent/good cosmetic results in 88-90% of patients [30-32]; while others showed a poor/fair cosmesis and/or moderate/severe fibrosis in 25-32% of patients with no conclusion on tolerance/efficacy of PBI versus conventional radiotherapy [33,34]. In the latter two papers the poor cosmetic/toxicity outcomes were related to the large breast volumes treated with relatively high doses in the postoperative setting.

Two studies with large cohorts showed encouraging results in terms of cosmesis/toxicity outcomes delivering a single high dose intra-operatively: Veronesi et al.[24] has reported with a median follow-up of 36 months only two patients with severe fibrosis among 1,800 women enrolled in the ELIOT trial, receiving a single 21Gy intra-operative RT(IORT) using electrons. The 4-year results of the TARGIT-A trial [35] conducted on 2,232 women from 28 centers in 9 countries showed that this approach had fewer RT-related side effects with respect to the conventional WBRT. A recent phase II study [36] reported 4 patients (9.5%) with G2 subcutaneous fibrosis and no ≥G3 side effects among 94 patients, older than 65 years, treated with exclusive PB IORT at 21Gy fraction, with a median follow up of 30 months. All these studies confirm the feasibility of IORT for treating early-stage breast cancer. However, many centers lack the specialized equipment and expertise required to deliver IORT.

The aim of the present study is to demonstrate the feasibility of an external beam radio-surgery approach as a more readily accessible alternative to IORT. In particular, the cine modality has been used to investigate the breast movement during treatment. This approach, based on the verification of the position of lead markers on the cine images, allows to verify that target movements due to breathing were trivial. To the best of our knowledge, there are no references in literature reporting dose constraints for organs at risk (OARs) to be used in the breast cancer treatment with single doses of photons. We used dose constraints reported in our previous paper [19], in principle based on reducing the maximum dose to the lowest part of the healthy tissue. Our study was designed to compare cosmetic results to WBRT(50Gy+boost), maintaining the same level of tumor control, assuming α/β = 10Gy, and it is the first reported experience of post-operative SSPBI by external photon beam RT as a part of BC treatment. Only one recent study[37] has tested the feasibility of a single fraction pre-operative PBI of 15Gy, resulting in a substantial dose reduction to the ipsilateral breast and a reasonable skin dose in comparison to their historical institutional controls treated with post-operative PBI. However, given the lack of iso-effective dose and the absence of clinical data no conclusion on outcome was provided.

Our results are indeed worse (63% of patients with unsatisfactory cosmetic outcome or ≥G2 late toxicity) than expected (29%) after conventional WBRT, leading to the premature closure of the study. Regarding the cosmetic outcomes, the WB mean dose resulted as a predictor of poor/fair outcome (p = 0.030). An increase of >G2 fibrosis(44%) was observed with respect to the 32.4%, reported at 10 years with a conventional fractionation in the European Organization for Research and Treatment of Cancer(EORTC) trial when the boost was treated[38]. In addition, our toxicity rate is higher than Chen et al.[32] reporting a moderate/severe fibrosis rate after external beam APBI of 25% after similar median follow-up, while it is in contrast with minimal local side effects reported in ELIOT/TARGIT studies. When investigating the late toxicity of APBI treatments it is critical to consider the differences in follow-up times: estimates suggest that 90% of the ultimate incidence (peak hazard) of fibrosis is expressed by approximately 4 years post-RT, depending on the grade of reaction and the intensity of the schedule[39]. Thus, our results do have the maturity of follow-up to draw reasonable conclusions about toxicity/cosmetic outcome.

Although adjuvant cytotoxic chemotherapy[40] has long been recognized as a risk factor for radiation-induced fibrosis after postoperative RT for breast cancer[41], we do not find any correlation with acute/late toxicity.

Understanding the impact of volume effect is important when considering fibrosis: larger volumes in our cohort seem to be predictive of ≥G1 or ≥G2 toxicity (P < 0.0001 and p = 0.0033,UVA). This correlation is confirmed for ≥G1 toxicity (p = 0.02, MVA) and becomes a trend for ≥G2 toxicity (p = 0.09,MVA).

Of note, the target volumes in our group (mean:108 cc, range:17-290 cc) were lower than those reported in other APBI series (the PTV evaluable mean:185- 296 cc, range:67-950 cc) this is probably due to the different surgical technique that involves the suture of the surgical breach without the formation of a lumpectomy cavity. Therefore, the use of a single dose is expected to be more toxic on normal tissues as well as effective on tumors, while lower volumes decrease the incidence of severe fibrosis(13%). Thus, the volume effect could explain the very limited moderate-severe fibrosis/fat necrosis, in 2/4.2% of patients, respectively, reported by Veronesi et al.[24]. In fact, with an applicator diameter of 4-6 cm and a dose of 21Gy prescribed at the level of 90% of the isodose, the expected V_18Gy _should range from 26 to 71 cc using a Novac (energies:7-9 MeV, applicators:40-60 mm) and from 34 to 85 cc using a Liac(energies:6-10 MeV, applicators:40-60 mm). The same observation may be suitable for TARGIT-A[35], where the Intra-beam device provides a point source of low energy X-rays(50kV maximum) at the tip of a 3.2mm diameter tube placed at the centre of a spherical tumor bed applicator. Thus, the volume effect should decrease the rate of toxic effects after a single high dose. In fact, in the TARGIT trial, doses of about 20Gy have been delivered using photon beam in a single fraction [35].

Another crucial issue is that the use of larger dose/fraction in APBI protocols presents specific radiobiologic concerns because of a possible reduction in reoxygenation and reassortment [41], requiring higher doses to maintain the same level of tumor control. The biological effects of a single dose under these circumstances might be different from those predicted from the LQ model. In particular, tumor hypoxic cells cannot reoxygenate, as occurs during fractionated RT, remaining radioresistant. In addition, some cells can be found in the "S phase" of the cell cycle (i.e. to be more radioresistant) and exhibit a greater level of survival compared to cells in "G2 phase" or mitosis [42]. Thus, a single fraction should require higher doses than those calculated with the LQ model.

Veronesi et al. [24] reported an actual local recurrence of 2.3% and an ipsilateral breast cancer of 1.2%. Whereas, in our series with a similar median follow-up the local recurrence was 1.6% (1/64) with no ipsilateral breast cancer, showing that local control rates compare favorably to other series after intra-operative delivery [24,36,37].

Regarding the BED using an α/β ratio of 10Gy our treatment schedule of 21Gy in single fraction is considered to be equivalent to a standard WBI of 50Gy+boost, while the other regimens using 3D-CRT or brachytherapy were equivalent to 50Gy.This leads to the following consideration; emerging data from several prospective randomized clinical trials [43-45] of hypofractionated WBI have recently estimated an α/β values of 4.6Gy for TCP and 3.4Gy for late changes in breast appearance (a surrogate of late toxicity). Adopting a low α/β ratio, BED values associated with our schedule were 116.9Gy and 157.0Gy for α/β ratios of 4.6Gy and 3.4Gy, respectively. These BEDs might have led to unexpected outcomes, worsening cosmesis/fibrosis, which could also be due to the larger volumes in our cohort (based on the additional margins to CTV used to reduce the rate of geographical missing), respect to IORT treatments with electron beams.

Considering the wide variability in treatment-related toxicity observed in our cohort, only in part explained by dosimetric factors, we aim at a closer scenario in which new factors, such as polymorphisms combined with dose parameters could better explain the different clinical radio-susceptibility [46].

## Conclusions

SSPBI in breast cancer patients after conservative surgery is feasible, however, based on our results and on the conflicting outcome reported in other PBI studies, we urge caution when applying this approach in the clinical practice. The single dose of 21Gy equivalent to a standard dose plus boost adopted in this prospective trial significantly increased the treatment-related toxicity in our cohort. However, this should not discourage the adoption of novel SSPBI approaches with lower equivalent doses as the advantages still outweigh the drawbacks.

## Abbreviations

3D-CRT: Three-dimensional conformal external beam radiotherapy; APBI: Accelerated PBI; BED: biological effective dose; CTCAE: Common Terminology Criteria for Adverse Events; CTV: clinical target volume;ELIOT: Electron Intraoperative Radiotherapy; G1, G2, G3: Grade 1,2,3; INBV: ipsilateral normal breast tissue; LQ: linear-quadratic; MVA: multivariate analysis; OARs: organs at risk; PBI: Partial Breast Irradiation; PTV: planning target volume; RT: radiotherapy; SSPBI: Single Shot 3D-CRT PBI; UVA: Univariate analysis; WB: Whole breast; WBI: Whole Breast Irradiation; WBV: Whole breast volume

## Conflict of interests

The authors declare that they have no competing interests.

## Authors' contributions

PP and GA conceived the study, coordination of the original study. PP, GA, LS supervised data analysis, interpreted the data analysis, drafted the manuscript and revised the final version of manuscript. AS, VL and GI performed the treatment plans, participated in the conceiving of the study and the implementation of the technique. AIF, CG, VP and SA data collection and patient management, and in drafting the final manuscript. All authors read and approved the final manuscript.
